# Self‐reported sexual dysfunction in patients with rectal cancer

**DOI:** 10.1111/codi.14907

**Published:** 2019-12-06

**Authors:** M. Sörensson, D. Asplund, P. Matthiessen, J. Rosenberg, T. Hallgren, C. Rosander, E. González, D. Bock, E. Angenete

**Affiliations:** ^1^ Department of Surgery Karlstad Hospital Karlstad Sweden; ^2^ Department of Surgery SSORG – Scandinavian Surgical Outcomes Research Group Institute of Clinical Sciences Sahlgrenska Academy University of Gothenburg Gothenburg Sweden; ^3^ Department of Surgery Region Västra Götaland Sahlgrenska University Hospital Gothenburg Sweden; ^4^ Department of Surgery Faculty of Medicine and Health Örebro University Örebro Sweden; ^5^ Department of Surgery Herlev Hospital University of Copenhagen Herlev Denmark

**Keywords:** Rectal neoplasm, sexual function, surgery, radiotherapy

## Abstract

**Aim:**

Patients with rectal cancer often experience sexual dysfunction after treatment. The aim of this study was to evaluate sexual function in a prospective cohort of patients regardless of treatment and tumour stage and explore what factors might affect sexual activity 1 year after diagnosis.

**Method:**

The QoLiRECT study (Quality of Life in RECTal cancer) is a prospective study on the health‐related quality of life in patients with rectal cancer in Denmark and Sweden. Questionnaires were completed at diagnosis and 1 year. Clinical data were retrieved from national quality registries.

**Results:**

Questionnaire data were available from 1085 patients at diagnosis and 920 patients at 1 year. Median age was 69 years (range 25–100). At diagnosis, 29% of the women and 41% of the men were sexually active, which was lower than an age‐matched reference population. This was further reduced to 25% and 34% at 1 year. Risk factors for sexual inactivity were absence of sexual activity prior to the diagnosis and the presence of a stoma. Women experienced reduced lubrication and more dyspareunia at 1 year compared with the time of diagnosis. In men, erectile dysfunction increased from 46% to 55% at 1 year.

**Conclusion:**

Sexual activity in patients with rectal cancer is lower at diagnosis compared with the population norm and is further reduced at 1 year. The presence of a stoma contributed to reduced sexual activity after operation. Sexual dysfunction was difficult to evaluate due to low sexual activity in the cohort. In men, erectile dysfunction is common.

**What does the paper add to the literature?**

The paper describes sexual dysfunction in an unselected cohort of patients with rectal cancer. Therefore, it reflects the effects of treatment in a wider perspective than previous literature. It highlights the problems encountered by patients with rectal cancer and shows that few patients receive supportive treatment.

## Introduction

Colorectal cancer is the third most common cancer in the world 1. There have been major improvements in both survival and local recurrence rates in the past decades due to improved treatment 2, 3. Today almost 80% are treated with curative intent. Treatment‐related morbidity, however, remains high and many patients have challenges related to their stoma as well as bowel, urinary or sexual function 4, 5, 6, 7, 8.

The incidence of sexual dysfunction among patients with rectal cancer varies in the literature from 5% to 88% 9, 10. This may be due to the lack of a common definition of sexual dysfunction which leads to difficulties in comparing results 10. Recently, an attempt has been made to develop a short seven‐item scale to evaluate female sexual function 11. However, this has yet to be tested in larger populations.

The cause of sexual dysfunction may be the surgery itself with potential nerve damage, especially during abdominoperineal excision for low rectal tumours 12, 13, 14. It has been suggested that minimally invasive techniques could reduce nerve damage and enhance the recovery of (uro)genital function but this has not been corroborated in larger studies 15, 16, 17.

Other possible risk factors for sexual dysfunction and low sexual activity are age, gender, neoadjuvant chemo/radiotherapy, advanced tumour stage and the presence of a stoma 14, 18, 19 as well as psychosocial factors such as depression and the patient’s social situation 20.

The primary aim of this study was to describe the prevalence of sexual dysfunction in an unselected patient cohort prior to treatment and 1 year after the diagnosis of rectal cancer. A secondary aim was to identify risk factors for low sexual activity 1 year after diagnosis.

## Method

### Patient selection and inclusion

The QoLiRECT (Quality of Life in RECTal cancer) study prospectively included 1248 patients recruited from 16 colorectal units in Denmark and Sweden between 2012 and 2015. All patients with a biopsy‐confirmed rectal adenocarcinoma within 15 cm from the anal verge, irrespective of tumour stage or treatment plan, who gave informed consent and were above 18 years of age were included. Details of the protocol have been described previously 21. During the study period, 3490 patients were registered in the Swedish and Danish national quality registries by participating centres. Of these, 2242 did not meet the inclusion criteria or were not asked to participate 22. A high response rate of the questionnaires was achieved through personal contact with all patients at inclusion as well as at follow‐up.

In short, the patients were included after diagnosis but prior to the start of treatment. Questionnaires were administered to each patient at inclusion and after 1, 2 and 5 years. The present analysis is based on the answers related to sexual function at inclusion and 1 year after diagnosis.

### The questionnaire

The questionnaire was developed using a process of in‐depth interviews with patients with rectal cancer, followed by a qualitative analysis, construction of questions, content validation in a multidisciplinary group and a face‐to‐face validation. This process has been used previously and some of the questions have been used in studies on patients treated for rectal cancer, prostate cancer and cervical cancer 22, 23, 24, 25, 26. The time frame for answers was set to 1 month to reduce the risk of recall bias 25, 27. For this study, we analysed questions relevant to sexual function.

### Reference population

We have previously evaluated the sexual function and sexual life within a population of 1078 randomly selected Swedish men and women with a median age of 63 years (range 31–90) 28. Initially, 3000 individuals randomly selected by the Swedish Tax Agency were contacted by letter and telephone. The recruited individuals completed a questionnaire similar to the study questionnaire and the results have been used for comparison in both this and other studies 22, 29.

### Sexual function

The questions were divided into male and female sexual function. Female sexual function focused on adequate/subjective physical reaction of sexual arousal described as genital swelling and lubrication during the last month. The presence of dyspareunia during intercourse or other sexual activity was also analysed. In the reference population, both deep and superficial pain were explored. This was combined into one variable for the analysis.

In men, erectile dysfunction was explored in the baseline questionnaire and the reference population using a yes/no question regarding weak erection. In the follow‐up questionnaire, the presence of dysfunction was derived from the responses to a question regarding the effect of erectile dysfunction on self‐esteem. Premature and retrograde ejaculation during the last month were evaluated in the reference population and at 1 year among the study population.

### Frequency of sexual activity

The frequency of sexual activity was obtained from the questions ‘How often have you had intercourse or other sexual activity in the last month?’, ‘Have you had thoughts about sex in the last month?’, ‘Has sex been important for you in the last month?’, ‘Have you felt sexually attractive in the last month?’ and the answers were dichotomized to ‘no activity’ *vs* ‘any activity’.

### Quality of sexual life

The quality of sexual life was derived from the questions ‘Are you satisfied with your sexual life’ and ‘Would you be distressed if the sexual problems/erectile dysfunction you experienced within the last month would remain the rest of your life?’ The ability to achieve orgasm was analysed in both sexes 1 year after diagnosis.

### Information regarding sexual dysfunction by healthcare givers

Whether patients received information regarding possible sexual dysfunction prior to treatment was evaluated by a yes/no question at baseline and after 1 year. To further address this issue, we also evaluated how many patients had sought medical help for sexual problems after treatment.

### Clinical data

Clinical data were retrieved from the national registries for colorectal cancer, in Sweden the Swedish Colorectal Cancer Registry (SCRCR) and in Denmark the Danish Colorectal Cancer Group (DCCG). Data on sex, age, American Society of Anesthesiologists classification (ASA), tumour stage [Union Internationale Contre le Cancer (UICC) classification], chemotherapy and/or radiotherapy, body mass index, type of surgery, technique (laparoscopic or open) and perioperative blood loss were collected. As some of the surgical and oncological data in the DCCG registry were lacking compared to the SCRCR, a clinical record form was developed at the study secretariat and filled out retrospectively by personnel at the participating Danish hospitals. Data regarding preoperative and postoperative chemotherapy, preoperative radiotherapy and type of surgery were collected. In the SCRCR, data were frequently missing on adjuvant chemotherapy. This information was added from the 1‐year follow‐up QoLiRECT questionnaire.

### Statistical analysis

Results are presented as numbers and percentages. Where the alternative ‘Not applicable, I have not had any sexual activity in the last month’ was possible, the percentages were calculated using only the sexually active patients.

Frequency of sexual activity and quality of sexual life as addressed by the four questions ‘Have you longed for sexual activity?’ (yes *vs* no), ‘Has sex been of importance for you?’ (yes *vs* no), ‘How often have you had intercourse or other sexual activity?’ (never *vs* once or more frequent) and ‘Have you been content with your sex life?’ (yes *vs* no), with a recall period of 1 month, were compared with the reference population in a gender‐ and age‐adjusted analysis using a log‐binomial regression with sex and age included as covariates. Results are presented graphically as prevalence estimates with 95% confidence intervals.

In the analysis of potential risk factors for absence of sexual activity after 1 year (‘How often have you had intercourse or other sexual activity in the last month?’), two types of variables were evaluated: those related to preoperative patient characteristics (sexual activity at diagnosis, age, sex, marital status and comorbidity) and those related to the intra‐operative and postoperative phase [UICC, preoperative chemoradiotherapy, radiation therapy, postoperative chemotherapy, type of surgery (anterior resection, abdominoperineal excision, Hartmann’s procedure, other or no operation performed), technique (laparoscopy *vs* open), stoma (‘Have you had a stoma during the last year?’) and the presence of a depressed mood (‘Would you consider yourself depressed?’, response options yes/don’t know *vs* no) at 1 year] 30. As the type of surgery is associated with the risk of having a stoma, the type of surgery and the question regarding stoma were combined into a single variable with three categories: (i) abdominoperineal excision (a stoma is mandatory); (ii) surgery/no surgery without a stoma; (iii) anterior resection/Hartmann’s procedure/other with a stoma.

Based on exploratory logistic regression analyses it was concluded that age, sex and marital status were associated with sexual activity both in the reference population and among patients at the time of diagnosis. It was further found that absence of sexual activity at diagnosis had a strong association with subsequent absence at 1 year. Therefore, in the analysis of activity at 1 year intra‐operative and postoperative variables were considered. Sexual activity at diagnosis was included as a covariate in the regression models. The variables were evaluated using simple and multiple logistic regression. As a means for selecting the risk factors with the strongest associations the purposeful selection strategy for logistic regression was used 31. The selection strategy consists of iteratively including and excluding variables from a multiple regression model based both on the explanatory power of the dependent variable as well as in the magnitude of the estimated regression coefficients between the different models. The SAS macro used to automatically perform the purposeful selection strategy used default values of the macro variables. The final step in the analysis involved replacement of missing values for the variables selected from the strategy using multiple imputations by chained equations 32 and the subsequent estimation and pooling of the final multiple regression model by using proc mianalyze in SAS. Local recurrence at 1 year was considered a potential risk factor, but as the number of patients with a local recurrence at 1 year remains low it was not considered.

## Results

**Figure 1:**
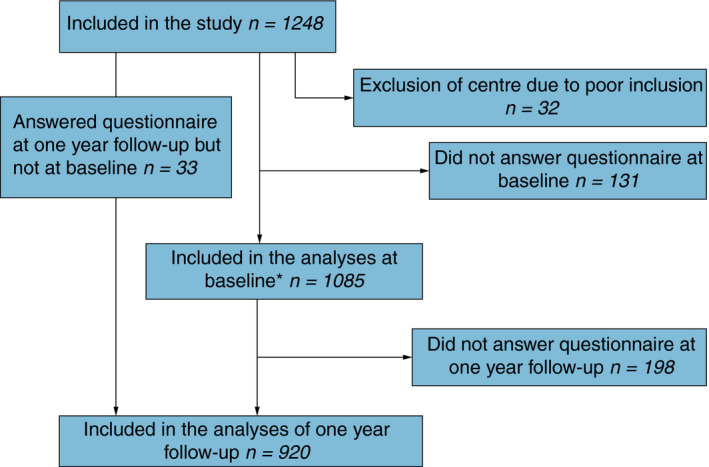
Flowchart of patients included in the analyses at baseline and the 1‐year follow‐up. Note that 33 patients answered the questionnaire at the 1‐year follow‐up but not at baseline. *Baseline questionnaire was distributed after diagnosis, prior to the start of treatment.

The questionnaire was completed by 1085 patients at inclusion and 920 patients after 1 year (Fig. 1). At 1 year, 49 patients had died. Among non‐responders at 1 year there were more patients with advanced tumours and patients without surgical intervention compared to responders 33. There were 63% men in the cohort and more men than women were in a relationship (Table 1). About half of the procedures were anterior resection and half were performed laparoscopically (Table 1). Compared with the reference population, the patients were similar regarding lifestyle factors such as smoking, physical activity and body mass index but had a lower alcohol consumption (Table 1).

**Table 1:** Baseline demography and patient characteristics of the QoLiRECT cohort and the reference cohort.

Variable	Category	QoLiRECT cohort				Reference cohort			
		Women (*n* = 392, 36%)	Men (*n* = 693, 63%)	Total (*n* = 1085)	Missing data	Women (*n* = 566, 52%)	Men (*n* = 512, 48%)	Total (*n* = 1078)	Missing data
Age	Median (range)	68 (25–93)	69 (38–100)	69 (25–100)	0	61 (31–90)	64 (32–90)	63 (31–90)	0
BMI (m/l^2^)	Median (range)	25 (22–28)	26 (24–29)	26 (23–29)	162	25 (17–47)	26 (18–49)	25 (17–49)	33
In a relationship	Yes	260 (67)	536 (78)	796 (74)	11	408 (72)	413 (81)	821 (77)	6
	No	127 (33)	151 (22)	278 (26)		154 (28)	97 (19)	258 (23)	
Current smoker	Yes	34 (9)	65 (10)	99 (9)	20	74 (13)	65 (13)	139 (13)	10
	No	353 (91)	613 (90)	966 (91)		489 (87)	440 (87)	929 (87)	
Alcohol intake	More than 16 glasses per week	4 (1)	34 (5)	38 (4)	46	116 (26)	126 (28)	242 (27)	186
	Less than 16 glasses per week	370 (99)	631 (95)	1001 (96)		329 (74)	321 (72)	650 (73)	
Physical activity (%)	Physically inactive	54 (14)	102 (15)	156 (15)	48	77 (14)	71 (14)	148 (14)	30
	Some light physical activity or more	324 (86)	557 (85)	881 (85)		470 (86)	430 (84)	900 (86)	
ASA classification	ASA I	96 (27)	139 (23)	235 (25)	133				
	ASA II	213 (61)	351 (58)	564 (59)					
	ASA III–IV	42 (12)	111 (18)	153 (16)					
Comorbidity*	Yes	234 (60)	427 (62)	661 (62)	12	310 (55)	271 (53)	581 (54)	1
	No	153 (40)	259 (38)	412 (38)		256 (45)	240 (47)	496 (46)	
Depression	Yes/I don’t know	87 (22)	94 (14)	181 (17)	9	86 (15)	63 (12)	149 (14)	10
	No	304 (78)	591 (86)	895 (83)		474 (85)	445 (88)	919 (86)	
Curative intention		375 (96)	637 (92)	1012 (93)	0				
Palliative intention		17 (4)	56 (8)	73 (7)	0				
UICC classification† (%)	0	6 (2)	12 (2)	18 (2)	140				
	I	114 (33)	164 (27)	278 (29)					
	II	80 (23)	144 (24)	224 (24)					
	III	110 (32)	186 (31)	296 (31)					
	IV	35 (10)	94 (16)	129 (14)					
Preoperative radiation therapy (%)	Yes	152 (39)	228 (33)	380 (35)	0				
	No	240 (61)	465 (67)	705 (65)					
Preoperative chemoradiotherapy (%)	Yes	63 (16)	154 (22)	217 (20)	0				
	No	329 (84)	539 (78)	868 (80)					
Postoperative chemotherapy (%)	Yes	145 (39)	246 (38)	391 (38)	64				
	No	226 (61)	404 (62)	630 (62)					
Operative technique (%)	Open	162 (46)	299 (50)	461 (48)	133				
	Laparoscopic	190 (54)	301 (50)	491 (52)					
Type of surgery (%)	Anterior resection	209 (56)	293 (45)	502 (49)	56				
	Abdominoperineal resection	108 (29)	223 (34)	331 (32)					
	Hartmann’s procedure	24 (6)	64 (10)	88 (9)					
	No intervention	19 (5)	48 (7)	67 (7)					
	Other‡	15 (4)	26 (4)	41 (4)					
Perioperative blood loss, ml (range)		150 (50–300)	250 (100–500)	200 (50–450)	166				

ASA, American Society of Anesthesiologists; BMI, body mass index; UICC, Union Internationale Contre le Cancer. Percentages are in parentheses.

* Comorbidity was characterized by a number of health conditions, including joint disorders, cardiovascular, neurological, pulmonary, renal, bowel and psychological conditions as well as diabetes and chronic pain, and was defined as the presence of at least one of these conditions.

† UICC is based on the pTNM classification.

‡ Other includes colectomy, Transanal endoscopic microsurgery (TEM), local excision, laparotomy without excision and unknown.

### Sexual function

More than half of the women with rectal cancer (59%–75%) chose the answer category ‘not applicable, I have not had sexual activity or been sexually aroused within the last month’ on one or several of the questions regarding sexual function. This was higher compared with the reference population (39%–45%). Women with rectal cancer generally had poorer sexual function compared with the reference population both at baseline and at 1 year. Dyspareunia increased almost twofold 1 year after diagnosis and there was a 50% increase of insufficient lubrication (Table 2).

**Table 2:** Sexual function.

Category	QoLiRECT cohort		Reference cohort
	Women (at baseline) (*n* = 392) (%)	Women (1 year after diagnosis) (*n* = 347) (%)	Women (*N* = 566)
Genital swelling during sexual stimulation the last month			
Yes	49/93 (53)	53/87 (61)	250/323 (77)
No	44/93 (47)	34/87 (39)	73/323 (23)
Not applicable	262/355 (74)	227/314 (72)	218/541 (40)
Missing	37	33	
Lubrication			
Sufficient	65/144 (45)	33/133 (25)	324/331 (98)
Insufficient	79/144 (55)	100/133 (75)	7/331 (2)
Not applicable	217/361 (60)	189/322 (59)	213/544 (39)
Missing	31	25	22
Dyspareunia			
No	78/103 (76)	42/80 (53)	219/296 (74)
Yes, at least a little	25/103 (24)	38/80 (47)	77/296 (26)
Not applicable	262/365 (72)	244/324 (75)	242/538 (45)
Missing	27	23	28

	Men (at baseline) (*n* = 693) (%)	Men (1 year after diagnosis) (*n* = 573) (%)	Men (*N* = 512)
Erectile dysfunction			
Yes	299/655 (46)	300/547 (55)	133/505 (26)
No	356/655 (54)	87/547 (16)	349/505 (69)
Don’t know		160/547 (29)	23/505 (5)
Missing	38	26	7
Retrograde ejaculation			
Yes		74/215 (34)	41/311 (13)
No		141/215 (66)	270/311 (87)
Not applicable		334/549 (61)	197/508 (39)
Missing		24	4
Premature ejaculation			
Yes		36/187 (19)	52/294 (18)
No		152/187 (81)	242/294 (82)
Not applicable		360/548 (66)	211/505 (42)
Missing		25	7

Among sexually active men retrograde ejaculation was three times as common in patients with rectal cancer at 1 year compared with the reference population. Erectile dysfunction was almost twice as common in men with rectal cancer at baseline (46%) compared with the reference population and was increased to 55% at 1 year (Table 2). Premature ejaculation did not differ between patients at 1 year and the reference population.

### Sexual activity

At baseline, about one‐third of the women had thoughts about sex, found sex important and had been sexually active within the last month. Results were similar at 1 year except that there was a 50% reduction in the number of women who included intercourse as a part of their sex life.

**Figure 2:**
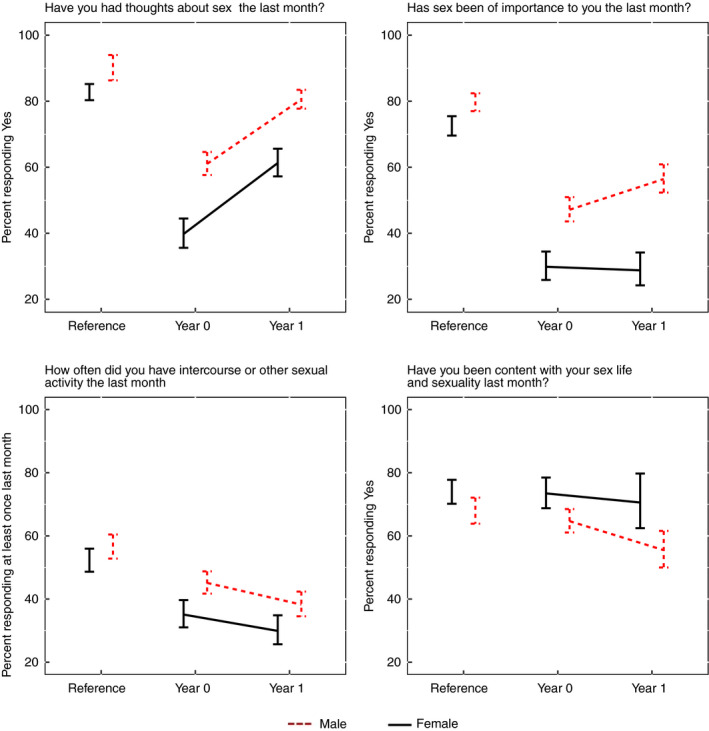
Age‐matched comparison with the reference population.

At baseline, almost 60% of the men had thoughts about sex, 50% found sex important and 41% had been sexually active within the last month. At 1 year, the number of men who had thoughts about sex and found sex important had increased compared to baseline, but the rate of patients with intercourse as part of their sex life was half compared to baseline. Compared with the age‐matched reference population, patients with rectal cancer generally had fewer thoughts about sex, found sex less important and had a lower sexual activity (Table 3 and Fig. 2).

**Table 3:** Sexual activity and quality of sexual life.

	QoLiRECT cohort				Reference cohort	
	Women (at baseline) (*n* = 392)	Women (1 year after diagnosis) (*n* = 347)	Men (at baseline) (*n* = 693)	Men (1 year after diagnosis) (*n* = 573)	Women (*N* = 566)	Men (*N* = 512)
Thought about sex the last month?						
Yes, at least a little	121/371 (33)	123/331 (37)	395/671 (59)	411/556 (74)	389/555 (70)	452/509 (89)
No	250/371 (67)	208/331 (63)	276/671 (41)	145/556 (26)	166/555 (30)	57/509 (11)
Missing	21	16	22	17	11	3
Has sex been important to you within the last month?						
Yes, at least a little	116/371 (31)	96/330 (29)	327/668 (49)	319/553 (58)	336/554 (61)	365/508 (72)
No	255/371 (69)	234/330 (71)	341/668 (51)	234/553 (42)	218/554 (39)	143/508 (28)
Missing	21	17	25	20	12	4
Have you felt attractive within the last month?						
Yes, at least a little	116/368 (32)	102/328 (31)	279/660 (42)	212/550 (39)	309/551 (56)	296/508 (58)
No	252/368 (68)	226/328 (69)	381/660 (58)	338/550 (61)	242/551 (44)	212/508 (42)
Missing	24	19	33	23	15	4
Have you refrained from sex due to risk of failure?						
Yes	35/371 (27)	50/330 (43)	79/667 (25)	115/555 (47)	51/550 (15)	78/506 (24)
No	96/371 (73)	66/330 (57)	235/667 (75)	126/555 (53)	284/550 (85)	249/506 (76)
N/A, no sexual activity	240/371	214/330	353/667	314/555	215/550	179/506
Missing	21	17	26	18	16	6
Have you taken an initiative to sex with your partner in the last month						
Yes	57/241 (24)	43/220 (20)	208/513 (41)	157/424 (37)	190/409 (46)	242/416 (58)
No	184/241 (76)	177/220 (80)	305/513 (59)	267/424 (63)	219/409 (53)	174/416 (42)
Not applicable, I have not had any sexual partner within the last month	125/366	105/325	153/666	123/547	141/550	91/507
Missing	26	22	27	26	16	5
What is your frequency of sexual activity within the last month						
At least 1–2 times per month	106/368 (29)	82/329 (25)	270/664 (41)	189/548 (34)	305/553 (55)	302/511 (59)
None	262/368 (71)	247/329 (75)	394/664 (59)	359/548 (66)	248/553 (45)	209/511 (41)
Missing	24	18	29	25	13	1
Is intercourse a part of your sexual life?						
Yes	158/356 (44)	64/326 (20)	361/646 (56)	140/544 (26)	329/546 (60)	315/507 (62)
No	198/356 (56)	262/326 (80)	285/646 (44)	404/544 (74)	217/546 (40)	192/507 (38)
Missing	36	21	47	29	20	5
Are you satisfied with your sexual life within the last month?						
Yes	240/322 (75)	81/116 (70)	411/639 (64)	167/297 (56)	395/523 (76)	331/496 (67)
No	82/322 (25)	35/116 (30)	228/639 (36)	130/297 (44)	128/523 (24)	165/496 (33)
Missing	70	22	54	32	43	16
Distress if sex problems/erectile dysfunction within the last month would remain the rest of your life?						
Moderate or much distress	54/304 (18)	71/295 (24)	270/521 (52)	226/304 (74)	121/535 (23)	173/502 (34)
No distress	10/304 (3)	13/295 (4)	76/521 (15)	20/304 (7)	38/535 (7)	48/502 (10)
Not applicable, no problem	240/304 (79)	211/295 (72)	175/521 (34)	58/304 (19)	376/535 (70)	281/502 (56)
Not applicable, no sexual activity/don’t know	52/356 (15)	24/319 (8)	117/638 (18%)	234/538 (43)	Not available	Not available
Missing	36	28	55	35	31	10
Has ability to achieve orgasm changed after treatment?						
Ability has not changed	–	87/123 (71)		126/332 (38)	282 (52)	324 (64)
Harder to achieve orgasm nowadays		30/123 (24)		139/332 (42)	36 (7)	32 (6)
I can’t achieve orgasm		6/123 (5)		67/332 (20)	18 (3)	13 (3)
Not applicable, could not before treatment		28/320 (9)		31/529 (6)		
Not applicable, have not tried to achieve orgasm		169/320 (53)		166/529 (31)	205 (38)	135 (27)
Missing		28		44	25	8
Afraid of embarrassment during sexual activity due to the stoma?*						
Yes, at least a little		38/67 (57)		93/150 (62)		
No		29/67 (43)		57/150 (38)		
Not applicable, I am not sexually active		111/178 (62)		196/346 (57)		
Missing		169		227		

* Number of patients with a stoma 1 year after diagnosis: women 170/331 (51%); men 343/557 (62%).

### Quality of sexual life

A majority of the women were satisfied with their sexual life before (75%) and after (70%) treatment). This was also similar to the reference population (74%). Before treatment, men with rectal cancer were similarly content with their sexual life compared with the reference population (64% *vs* 67%), but still less satisfied than women. At 1 year, men with rectal cancer were less satisfied than before treatment (56%) and 74% reported much distress if their sexual dysfunction would remain, compared with 52% before treatment and 34% in the reference population. Women with rectal cancer had low distress at baseline but similar distress to the reference population at 1 year (24% *vs* 23**%)** (Table 3 and Fig. 2).

In the majority of women, the ability to achieve orgasm did not change over time, but in men 42% experienced problems achieving an orgasm 1 year after diagnosis (Table 3).

### Risk factors for low sexual activity

Of all evaluated potential risk factors for low sexual activity after 1 year (Table 4), being sexually inactive at diagnosis and the presence of a stoma were identified by the regression selection strategy (Table 5).

**Table 4:** Logistic regression analysis of potential risk factors for absence of intercourse last month 1 year after diagnosis.

Variable	Category	Intercourse frequency last month		Comparison	Bivariate regression*		Multiple regression	
		Once or more frequent, *n* = 271 (31)	Never, *n* = 606 (69)		OR (95% CI)†	*P* value	OR (95% CI)†	*P* value
Intercourse frequency last month, at diagnosis	Once or more frequent	202 (64)	114 (36)	Never *vs* once or more frequent	16.67 (11.11; 25.0)	< 0.0001	16.67 (11.11; 25.0)	< 0.0001
	Never	49 (10)	454 (90)					
	Missing	20	38					
UICC	UICC 0	2 (14)	12 (86)					
	UICC I	76 (33)	153 (67)	I *vs* II	1.18 (0.71; 1.99)	0.5205	1.10 (0.64; 1.89)	0.7371
	UICC II	63 (35)	116 (65)	I *vs* IV	0.86 (0.44; 1.686)	0.6611	0.72 (0.30; 1.72)	0.4583
	UICC III	66 (28)	172 (72)	II *vs* III	0.60 (0.36; 0.99)	0.0495	0.48 (0.25; 0.91)	0.0255
	UICC IV	26 (29)	64 (71)	II *vs* IV	0.73 (0.36; 1.45)	0.3664	0.66 (0.28; 1.57)	0.3425
	Missing	38	89					
Preoperative CRT	No	216 (31)	475 (69)	No *vs* yes	0.75 (0.48; 1.18)	0.2110	0.75 (0.38; 1.48)	0.4123
	Yes	54 (29)	131 (71)					
	Missing	1						
Preoperative RT	No	174 (32)	378 (68)	No *vs* yes	0.88 (0.61; 1.28)	0.5092	0.98 (0.61; 1.58)	0.9384
	Yes	96 (30)	228 (70)					
	Missing	1						
Postoperative CT	No	153 (29)	366 (7)	No *vs* yes	1.05 (0.73; 1.52)	0.8004	1.70 (0.96; 3.00)	0.0668
	Yes	117 (3)	238 (67)					
	Missing	1	2					
Surgical technique and colostomy	1: APE	78 (28)	196 (72)	1 *vs* 2	1.66 (1.08; 2.55)	0.0212	1.57 (0.94; 2.61)	0.0846
	2: AR, other or no surgery, no stoma	128 (38)	206 (62)	1 *vs* 3	0.78 (0.46; 1.30)	0.3359	0.83 (0.46; 1.48)	0.5886
	3: AR, Hartmann, other or no surgery, stoma yes	52 (25)	157 (75)	2 *vs* 3	0.47 (0.29; 0.77)	0.0026	0.53 (0.31; 0.91)	0.0164
	Missing	13	47					
Technique	Open	108 (29)	264 (71)					
	Laparoscopy	138 (34)	264 (66)	Laparoscopy *vs* open	0.89 (0.61; 1.29)	0.5369	1.03 (0.67; 1.57)	0.9027
	Missing	25	78					
Would you call yourself depressed?	No	238 (32)	515 (68)	No *vs* yes/don’t know	0.88 (0.50; 1.55)	0.6542	0.77 (0.39; 1.50)	0.4438
	Yes/don’t know	31 (26)	87 (74)					
	Missing	2	4					

APE, abdominoperineal excision; AR, lower anterior resection; CRT, chemoradiotherapy; CT, chemotherapy; Hartmann, Hartmann’s procedure; RT, radiotherapy; UICC, Union Internationale Contre le Cancer.

* Intercourse frequency at diagnosis is included as a covariate.

† Modelling the probability of no intercourse in the last month.

**Table 5:** Multiple logistic regression analysis for the variables chosen by the selection strategy*.

Variable	Comparison	Without imputations		With imputations	
		OR (95% CI)	*P* value	OR (95% CI)†	*P* value
Intercourse frequency last month, at diagnosis	Never *vs* once or more frequent	16.67 (11.11; 25.0)	< 0.0001	14.29 (9.09; 20.00)	< 0.0001
Surgical technique and colostomy‡	1 *vs* 2	1.66 (1.08; 2.55)	0.0212	1.58 (1.05; 2.38)	0.0273
	1 *vs* 3	0.78 (0.46; 1.30)	0.3359	0.84 (0.51; 1.38)	0.4856
	2 *vs* 3	0.47 (0.29; 0.77)	0.0026	0.53 (0.33; 0.84)	0.0082

APE, abdominoperineal excision; AR, lower anterior resection; Hartmann, Hartmann’s procedure.

* Modelling the probability of no intercourse in the last month.

† Pooled estimates based on multiple imputations by chained equations.

‡ 1, APE; 2, AR, Hartmann, other or no surgery, no stoma; 3, AR, Hartmann, other or no surgery, stoma yes.

### Information and treatment for sexual dysfunction

Most patients did not remember receiving any information regarding possible sexual dysfunction after treatment at the 1‐year follow‐up. Women experienced to a larger extent that they had not been informed. Only 14% of the men and 4% of the women had sought help for their sexual dysfunction (Table 6).

**Table 6:** Information and medical assistance.

Category	Women (at baseline) (*n* = 392)	Women (1 year after diagnosis) (*n* = 347)	Men (at baseline) (*n* = 693)	Men (1 year after diagnosis) (*n* = 573)
Information about sexual dysfunction after treatment for rectal cancer				
Yes	59/378 (16)	67/326 (21)	189/675 (28)	148/546 (27)
No	319/378 (84)	249/326 (76)	486/675 (72)	377/546 (69)
Don’t remember/know		10/326 (3)		21/546 (4)
Missing	14	21	18	27
Sought medical support after treatment?				
Yes		8/210 (4)		60/437 (14)
No		129/210 (61)		293/437 (67)
Not applicable, I have no problems		73/210 (34)		84/437 (19)
Not applicable, I have not had any sexual activity within the last month		110/320 (34)		106/543 (20)
Missing		27		30

## Discussion

This study shows that sexual activity is low in an unselected cohort of patients with rectal cancer at the time of diagnosis and even lower 1 year later. It is also obvious that many patients experience that they have not received information regarding the likely effects of treatment on sexual function.

In our study, sexual dysfunction was not as pronounced as previously reported at 1 year 9, 10, 14, 29. Just as described by a recently developed short form for the evaluation of female sexual activity in patients with rectal cancer 11, we found that insufficient lubrication and swelling are also common problems both at diagnosis and after 1 year. Interestingly, dyspareunia was similar in female patients at baseline and the reference population although this almost doubled after 1 year, suggesting a clear negative effect from the treatment.

Even though the prevalence of dysfunction was high it is evident that most women were content with their sexual life. This highlights that satisfaction may be related to other issues, such as the level of expectation. Possibly, the high level of retained ability to achieve an orgasm in women after treatment could be a part of the reason for the degree of satisfaction in this group. Interestingly, men were less satisfied and they also had twice as frequent problems achieving an orgasm. Both men and women felt insufficiently informed about their possible sexual dysfunction. Still, women were fairly pleased, indicating that lack of information did not affect their experience of their sexual life.

The twofold higher prevalence of erectile dysfunction at diagnosis among patients with rectal cancer compared with the reference population indicates that our patient cohort is somewhat older and that erectile dysfunction may also be related to other factors than nerve damage and the surgical procedure. Interestingly, 95% of the men answered the questions regarding erectile dysfunction although other answers indicated that not all of these patients were in fact sexually active. Our figures on erectile dysfunction are similar to or lower than previously reported 8, 9.

The risk of erectile dysfunction should be addressed when counselling patients, and our study indicates that information to patients remains insufficient. Not more than 28% of the men and 16% of the women experienced that they had been informed about the possible side effects on their sexual function at diagnosis. This was similar after 1 year and very few patients had sought medical treatment. There are data indicating that men with post‐treatment sexual dysfunction may benefit from sildenafil 34 and that this should be initiated soon after treatment. For women, assistance with lubrication and counselling regarding dyspareunia could improve their sexual function 35. Our data show that we need to raise awareness among caregivers that patients do not receive sufficient information.

Sexual activity was lower in patients with rectal cancer compared with the reference population, both at baseline and at the 1‐year follow‐up. This is not surprising as a Dutch study that focused on patients and their partners at diagnosis revealed a reduced sexual activity and quality at diagnosis 36 and there are other studies that have found similar sexual frequency to ours in patients after treatment 14. Still, there are other studies that indicate that our patients were less sexually active at baseline than previously reported 8, 11. In part, this may be due to the slightly younger cohort in these studies but it may also be due to recall bias in cross‐sectional cohorts 11.

Sexual dysfunction after rectal cancer treatment is a multifactorial problem with a broad spectrum of associated risk factors 20, 37. Psychological factors such as libido, arousal, body image and self‐esteem may be important 11, 38. The presence of a stoma seems to affect sexual life for many patients, and improved counselling both before and after surgery by stoma care therapists as well as other healthcare personnel may help these patients.

A strength of this study is the prospective, unselected, multicentre design. Patients have been included regardless of curative or palliative intent. We previously compared the cohort to all patients admitted to the including hospitals, and it is a representative cohort of daily clinical activity 22. The large cohort together with good response rates at baseline and at 1‐year follow‐up are also considered strengths. The multicentre design with almost no exclusion factors gives high external validity. The reference population is a strength, as it was collected during the same time frame as the study. We used a recall period of 1 month in the questionnaire and this may cause a bias regarding sexual function as it is not evaluated in patients with low sexual frequency. However, a longer recall period is also problematic as it may introduce recall bias. It is important that patients can find a response option that applies to them, and thus the answer ‘Not applicable, I have not had any sexual activity’ has a value in itself.

In conclusion, regardless of gender, low sexual activity 1 year after diagnosis and treatment for rectal cancer is not only related to the diagnosis but also to factors that are unrelated to the disease. The presence of a stoma seems to be of importance and measures to facilitate patients with permanent or temporary stomas regaining their sexual activity must be a focus. Preoperative information about sexual dysfunction after treatment for rectal cancer should be improved.

## Acknowledgements

The Swedish Research Council, grant numbers 2012‐1768 and 2017‐01103; the Swedish Cancer Society CAN 2013/500 and CAN 2016/509; the Swedish Society of Medicine; the Gothenburg Medical Society; the Healthcare Sub‐committee, Region Västra Götaland; ALF grant ALFGBG‐526501, ALFGBG‐136151, ALFGBG‐493341 and ALFGBG‐716581 ‘Agreement concerning research and education of doctors’; Anna‐Lisa and Bror Björnsson Foundation; Assar Gabrielsson Foundation; Mary von Sydow Foundation; Ruth and Richard Julin’s Foundation, Lion’s Cancer Research Foundation of Western Sweden.

## Conflicts of interest

None of the authors has any conflicts of interest.
